# Potassic Nitrate in Scarlatina

**Published:** 1877-04

**Authors:** F. B. Eisen Bockius

**Affiliations:** Consulting Physician to North Star Dispensary


### THE NITRATE OF POTASSIUM IN THE TREATMENT OF SCARLATINA.

By F. B. EISEN BOCKIUS, M. D., LL. B.,
(Consulting Physician to North Star Dispensary.)

At the present time, when the concentrated gaze, not only of the profession, but of the whole public, is turned to the prevailing epidemic of scarlet fever, it seems to me both wise and fitting that medical practitioners should exchange, in some tangible form, the record of results of different modes of treatment in this disease; and in pursuance of this belief I propose to submit my own experience in the use of the nitrate of potash, and the logical basis for such use, together with those points of collateral treatment which stand prominently associated with the success of that experience.

As a preposition to my essay, I will be pardoned for saying that, in my opinion, much valuable time and effort have been expended in ascertaining the province of new remedies, that would better have been rewarded in extending and developing the well known qualities of an old one.

I shall assume as tenable:

1. That the simplest and mildest of medicinal agents (in necessary properties equal), is most preferable.

2. That scarlatina is a zymotic disease,

(*a*). Characterized by a hyperplastic condition of the circulating fluids;

(*b*). That the primary danger arises from the direct influence of the septic poison on the blood, lymph and chyle; and may be measured by the harshness of the febrile symptom;

(*c*). That the secondary danger is due to inflammations of the serous and mucous membranes, and adenitis (sequelæ); which have their origin in the primary corruption of the nutritive fluids.

And in favor of the use of the potash salt, I shall urge a priori:

1. That of all the quasi-specific agents recommended in the zymotic fevers none possesses the accidents of popular employemnt, harmlessness and mildness of administration, in as great a degree as the nitrate of potash; while some, notably carbolic acid and the sulpho-carbolates, are violent local and cerebro-spinal irritants.

2. Whatever may be the theory of scientific men, based on the disagreeable odor of the remedies, experience in the arts firmly inculcates that no antiseptic has more honest claims for efficiency in destroying the germ of putrefaction and arresting fermentation, without impairing the nutrient properties of the material acted upon, than the subject of this article; experiments conducted in the field of the microscope demonstrate the rapid cessation of life in rudimentary animal and vegetable structures, when subjected to the influence of this drug. What then was my surprise to find that not one of our standard works on materia medica,or therapeutics devotes even a single word to this quality of the nitrate which has rendered it invaluable for the preservation of meats in the commercial world.

(*a*). All works on therapeutics agree in ascribing to this salt, in an eminent degree, the property of rendering the blood aplastic in the living, and maintaining its fluidity in the dead subject; while through the partial decomposition of the drug, in the circulation, and the consequent liberation of a portion of its abundant oxygen, the whole mass is rendered arterial, retaining its hue even after death. As plasticity of the blood seems an essential of rheumatism, which is a common sequela of scarlatina, the indication for the employment of the nitrate is plain.

(*b*). Practical therapeutists also agree in conceding to this salt the power of reducing the force and frequency of the pulse, and lowering the temperature of the body; requirements which are essential in this disease, and which are possessed by no other antiseptic.

(*c*). If the nitrate of potash is an antiseptic then, in so far as the specific poison, by contaminating the blood and lymph, is instrumental in developing serous, mucous, renal and lymphatic inflammations, the effectual eradication of the germ and its arrested reproduction by means of this remedy, would measurably ameliorate these local symptoms; while if employed before their development it should prevent their occurrence. In the case of the principal gland liable to disease—the kidney—it should afford almost total immunity, both through its sedative action on the circulation, reducing renal congestion, and by its peculiar diuretic influence, being that of a stimulant to the functional activity of the secreting cell, instead of causing turgescence of the afferent vessels, as do the irritants; indeed, I know of no single remedy so certain of affording relief in renal suppression, from whatever cause, as the nitrate of potash.

With this theoretical showing in favor of my subject, I shall briefly consider its utility as an antiseptic in zymotic diseases in the light of experience. When I commenced the use of this drug, in the treatment of scarlet fever, I was not aware that it had ever been employed, or even recommended in the septic diseases; a hasty search through such works as were readily accessible, revealed but one such instance, being that found in Kost's Mat. Med. and Therapeu., p. 242, where mention is made of the great success of Dr. Stottsberry in the treatment of yellow fever by this medicine; and that of Dr. Stevens, who used it very extensively and with pre-eminent success in combatting the putrescent (zymotic?) fevers of British India; both experiments of quite recent date. To these facts I must be permitted to add my own observation during the present epidemic, in the early part of which I resorted to the ammon. carb. and belladonna treatment, with indifferent success, the disease not being dangerous, but the sequelae troublesome. Since employing the nitrate in scarlet fever, I have kept record of a large proportion of cases for the purpose of determinining whether the effect of the medicine was merely to delay the development of such sequelae as renal and lymphatic adenitis, or to completely obviate them with an opinion in favor of the latter; this record comprises one hundred and twenty-three cases, in which there were three deaths, all of the malignant type, two of which had been medicated injudiciously by a notorious quack, which had, however, but little influence on the result; cases so singularly free from disagreeable complications that I am compelled to believe that the harmlessness of the disease is due to the peculiarity of the treatment. The dose in which I have administered the remedy to children under 8 years of age, has varied from one to five grains, repeated every second or third hour during the first day and night, and every third or fourth hour (depending on the force of the circulation) afterward, until the normal action of the heart announces the cessation of septic irritation; the combinations in which I have used it, have been ipecac, opium and spt. nitre. Where decided evidences of weakness occur, I have prescribed quinia and iron; or better, if there be much emaciation, calcii phosph., pepsina and ol. morrhuæ in emulsion.

In those instances where the anginose symptom is pronounced, it is my practice to direct fat salt pork, sliced thin, stitched to a flannel, and sprinkled with powdered gum camphor, to be applied sedulously to the throat, until the impetiginous eruption, it is sure to produce, becomes unbearable; I do not favor the internal applications so frequently and harmfully made, where a frail, nervous child, suffering intensely from faucial inflammation, is harshly seized, its jaws pried open with a spoon, and while the little one struggles with its remaining strength for the necessary breath, the humane (?) physician shuts his eyes as if in sympathy, and thrusts a barbarous sponge probang, again and again, against the fragile membrane, tearing the immature cicatrices asunder, and leaving the whole faucial surface one bleeding sore; and then the intelligent (?) practitioner pathetically asks why his patients die, as if to live under such treatment were a pleasure. Whatever applications are to be made to the throat internally, should be conveyed by a camel's hair pencil; the combination I have found most useful, in the few cases I have had occasion to employ it, consists of

| | |
|---|---|
| Acidi carbolici | grs. x—xx |
| Liq. ferri subsulph. | Min. x—xx |
| Potass. chloratis | ʒss |
| Aq. destil. | fʒi |

to be applied only by the physician himself.

Even where the throat symptoms are but slight, the officinal linimentum camphoræ should be embrocated, and the throat protected by a flannel; demulcent drinks to be freely allowed; temperature of the apartment to be kept at 70 °, and the air moist; no baths are to be given at a lower temperature than that of the surface, until the eruption has faded. Under this treatment, faithfully executed, the period of the eruption is shortened at least one day, and the sequelæ, which commonly follow in its track, are almost unknown; in my experience 89 per cent., of those who had no complication when I first saw them, escape all complications. In closing, I wish to say that, however severe may be the local disturbance at the commencement of the disease, and whether peritonitic, urinary or gastro-intestinal, it will more surely and certainly disappear beneath the full, free and fearless administration of this salt, than under the usual treatment for idiopathic affections.

